# Identification and classification of repeated whistle types from free-ranging rough-toothed dolphins (*Steno bredanensis*)

**DOI:** 10.1038/s41598-026-44853-2

**Published:** 2026-03-20

**Authors:** Laura Redaelli, Vincent M. Janik, Filipe Alves, Julie N. Oswald, Marc Fernandez, Eliette Hamard, Laela S. Sayigh, Manuel E. dos Santos, Ana Dinis, Francesco Caruso

**Affiliations:** 1 MARE – Marine and Environmental Sciences Centre, ARNET – Aquatic Research Network, Regional Agency for The Development of Research, Technology and Innovation (ARDITI), Funchal, Madeira, Portugal; 2 Faculty of Life Sciences, University of Madeira, Funchal, Portugal; 3 MARE – Marine and Environmental Sciences Centre, ISPA – Instituto Universitário, Lisbon, Portugal; 4 Sea Mammal Research Unit, Scottish Oceans Institute, School of Biology, University of St. Andrews, Fife, UK; 5 Biology Department, Woods Hole Oceanographic Institution, Woods Hole, MA USA; 6 Department of Marine Animal Conservation and Public Engagement, Stazione Zoologica Anton Dohrn, Naples, Italy

**Keywords:** Acoustic, Communication, Madeira Island, Marine mammal, Delphinid, Neural network, Ecology, Ecology, Evolution, Neuroscience, Zoology

## Abstract

Acoustic communication is vital for marine mammals. Many delphinids rely on frequency-modulated tonal signals – known as whistles – used during social communication. Among these, individually distinctive “signature whistles” have been extensively studied in bottlenose dolphins (*Tursiops truncatus*) over the past 60 years. These stereotyped whistles convey individual identity and motivational information. Recent studies have identified similar stereotyped whistles in other delphinids, including a rehabilitated rough-toothed dolphin (*Steno bredanensis*). However, the function, occurrence, time–frequency characteristics, and degree of stereotypy of whistles in free-ranging individuals of this species remain unexplored. This study analysed acoustic recordings from three encounters with rough-toothed dolphins in summer 2023 off Madeira Island (Eastern North Atlantic). Of 4928 whistles analysed, 1015 were identified as repeated whistles and classified into 25 categories based on frequency modulation contours using visual inspection. Visual classification was verified using an inter-observer reliability test and compared to results from an unsupervised neural network (ARTwarp), employing both sequential and global analyses. The results revealed repeated contour types, with the degree of stereotypy varying across categories and generally lower than that reported for other well-studied delphinid species. Like other delphinids, rough-toothed dolphins exhibit a fission–fusion social structure, where repeated signals may help maintain group cohesion, potentially functioning as individual- or group-level calls. Future research incorporating detailed contextual and behavioral information will be crucial to investigate the function, occurrence and social relevance of repeated calls in rough-toothed dolphin whistle repertoires.

**Supplementary Information:**

The online version contains supplementary material available at 10.1038/s41598-026-44853-2.

## Introduction

In the animal kingdom, communication comes in different forms. Terrestrial animals utilize sensory channels such as vision, audition, olfaction, or touch to share information intra– or inter–specifically^1^. The oceanic environment, however, poses significant challenges to using two of these senses due to an absence of olfactory stimuli and light attenuation and scattering^2,3^. Therefore, many marine animals have adapted to rely primarily on acoustic cues to transmit information at a distance^4^. Among cetaceans, the toothed whales (odontocetes) use various sound categories to carry out their vital and social functions^5,6^. One such category consists of narrowband frequency-modulated tonal sounds, referred to as *whistles*, that are used for communication. Many studies have shown that these whistles appear to be essential for maintaining social cohesion and coordinating group activities^7–9^.

Toothed whales display various examples of complex social dynamics and communication systems, in which the social context of each animal is a strong driver of its vocal behaviour^6^. For example, the vocal repertoires of matrilineal groups and pods of killer whales (*Orcinus orca*) include stereotyped calls that are shared among group members^10,11^. In contrast, many species of smaller delphinids live in fission-fusion societies, in which groups are relatively transitory and fluid, and individuals produce specific calls to mediate interactions with conspecifics^12^. Within these dynamic social groups, conspecifics form long-lasting associations for several purposes, such as mating, foraging, and nursing^12^. The development of individually distinctive acoustic calls can help maintain socially specific relationships by broadcasting identity over relatively long distances^13–15^ in such groups. These individually distinctive acoustic signals, called “signature whistles”, were first identified in captive common bottlenose dolphins (*Tursiops truncatus*) in 1965, and have since been demonstrated to convey individual identity to conspecifics^16–18^.

Since 1965, many studies have been focused on bottlenose dolphin signature whistles in both wild and captive settings (see^19^ for an overview). While individual signature whistles can vary in certain characteristics, such as duration, they retain a consistent overall frequency modulation pattern or contour, which makes them recognizable^20^. Most research on signature whistles has focused on bottlenose dolphins, largely due to their widespread and coastal distribution^19^ and their prevalence as the most frequently kept delphinid species in captivity^21^. However, evidence of stereotyped whistles has been found in several other delphinid species, including the short-beaked common dolphin (*Delphinus delphis*^22–24^, Pacific white-sided dolphin (*Lagenorhynchus obliquidens*)^25^, Atlantic white-sided dolphin (*Lagenorhynchus acutus*)^24^, Atlantic spotted dolphin (*Stenella frontalis*)^26^, spinner dolphin (*Stenella longirostris*)^27^, Guiana dolphin (*Sotalia guianensis*)^28,29^, and Indo-Pacific humpback dolphin (*Sousa chinensis*)^30–32^. The function of these stereotyped whistles is currently unknown for most of these species. The study of stereotyped whistles in dolphin species remains limited due to the difficulty of accessing and properly recording offshore populations. Some exceptions arise through opportunistic situations, such as recordings of stranded or rehabilitated individuals, which provide rare opportunities to investigate whistle production in species that are otherwise challenging to study. Evidence suggestive of signature whistles has been found in several species under such circumstances (e.g.^19,22,30,31^). An example is provided by Ramos et al.^33^, who recorded a rehabilitating rough-toothed dolphin (*Steno bredanensis*) following a live stranding. Over eight days, these authors performed health assessments and daily vocal recordings. Using spectrogram analysis and the SIGnature IDentification (SIGID) method, they analyzed temporal whistle patterns to identify repeated contours that occur in a similar bout structure to signature whistles in bottlenose dolphins^34^. While investigations on well-studied species such as bottlenose dolphins provide valuable insights into repeated stereotyped whistles, their functional significance may not be directly extrapolated to less-studied species such as rough-toothed dolphins, for which comparable evidence remains limited.

The presence of repeated call types in a vocal repertoire does not necessarily imply signature whistle use. In many animal species, including odontocetes like short-finned pilot whales (*Globicephala macrorhynchus*), repeated stereotyped call types have been documented that are shared within social groups or are context-specific but do not appear to be individually distinctive^35^. Therefore, while repeated whistle types produced by isolated individuals may resemble signature whistles in their stereotypy and repetition, it remains unclear whether these are individually distinctive, shared among subgroup members, or occur at a wider level^4^. Furthermore, recording a single isolated individual does not provide definitive evidence for the presence of signature-based communication. In fact, in some species, individuals may repeatedly produce the same call when isolated, even if the call is not unique to that individual^36^.

Rough–toothed dolphins are distributed globally, inhabiting tropical, subtropical and temperate waters^37^. Because of their oceanic range, knowledge of their ecology, social and acoustic behaviour remains limited and is primarily derived from a few regions, such as near oceanic islands in the Pacific^38,39^ and along the coast of Brazil^40,41^. Overall, the species remains poorly understood, and in the North Atlantic, studies on their acoustic behaviour are limited to two analyses of data collected in the Canary Islands^42,43^. The few available acoustic studies indicate that rough-toothed dolphins produce clicks and tonal sounds, similar to other delphinids, with whistles that are characterised by frequency steps, temporal breaks and limited frequency modulation^40,43–45^. Recent studies into their social structure suggest that these dolphins form complex fission–fusion societies^46^. Field observations in the Canary Islands indicate that groups typically consist of several tight subunits, each composed of two to seven individuals swimming in close synchrony and often maintaining body contact, while the overall group remains spatially dispersed^47^. Distances between subunits can reach tens of meters, and group configurations are fluid, with subgroups merging or separating over time^47^. Such dynamic social structure may have promoted different processes of acoustic identity signalling, ranging from individual to group levels, raising intriguing questions about whether repeated calls function as signature whistles or as shared group identifiers, as proposed in other odontocetes^11,48^. In this study, the occurrence of repeated stereotyped whistles in free–ranging rough–toothed dolphins recorded off the coast of Madeira Island was assessed and characterised for the first time. The integration of visual and automatic methodologies allowed us to categorize contour similarities of whistles while reporting on their temporal patterns of occurrence and acoustic characteristics.

## Methods

### Study area and data collection

Underwater acoustic recordings of free–ranging rough–toothed dolphins were collected during the summer of 2023 off the south coast of Madeira Island (Portugal, Eastern North Atlantic) from dedicated surveys in the Rigid Inflatable Boat (RIB) R/V Observatório I.

We used a Soundtrap 300HF recorder (Ocean Instruments Inc., New Zealand) with a sampling rate of 288 kHz in continuous recording mode. This instrument has a frequency range of 20 Hz – 150 kHz ± 3 dB, and sensitivity of − 201 dB re V/µPa (high gain setting with a maximum sound pressure level of 174.7 dB re 1 µPa peak to peak before clipping). The recorder was attached to a nylon rope with a swivel and deployed at 7 to 10 m depth, when at least part of the group was within 100 m of the boat. The recordings were conducted from the vessel with the engine turned off. If repositioning of the vessel was necessary, usually due to animal movements, the hydrophone was retrieved, the recording paused and then re–deployed once a better position was reached. No other cetacean species were observed in the area during these sampling events.

For each recording, additional data were collected on the initial sighting location, group composition, and nearby vessels’ presence. During the sampling event, photographs of individual dorsal fins were taken using digital cameras (Canon EOS 7D Mark II; 400 millimetres lenses) before, during and/or after the recording session, when the animals were in close proximity to the vessel. These photographs contribute to the photographic–identification (hereafter photo–ID) catalogue of the species in the area (available online at https://happywhale.com) and were used to identify distinctive individuals, allowing assessment of group composition and the potential recurrence of individuals across encounters. Photo–ID followed standard procedures^49^, and only high-quality images of well–marked individuals were used (as per^50^).

### Acoustic data analysis

This study applied both visual and automatic categorization of spectrograms. Visual classification was performed first and served as a baseline for comparison, enabling an assessment of how well the categories generated by the automatic method aligned with those identified through visual inspection.

**Fig. 1:**
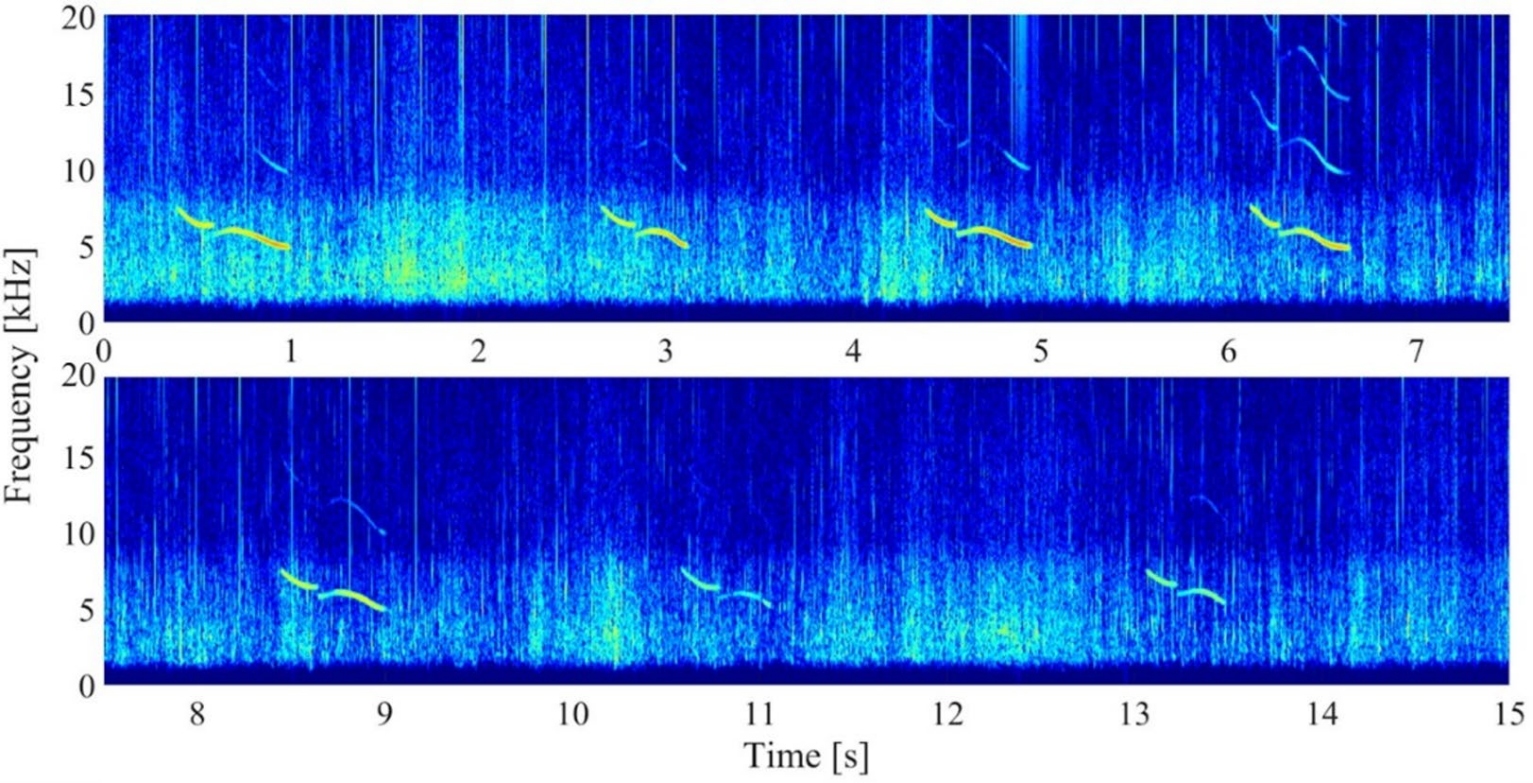
Example of the spectrogram view of a repeated stereotyped whistle bout containing seven consecutive signals in a 15-second sequence. Spectrograms computed in MATLAB for illustration purposes with the following parameters: FFT = 4096, Hamming window, overlap = 90%, frequency range = 0–50 kHz (then cropped to 20 kHz), time window = 7.5 s. A bandpass filter (2–25 kHz) was applied prior to spectrogram computation, and amplitude values were clipped below − 120 dB to enhance contrast.

Whistles were considered following their definition as narrow-band tonal signals with a minimum duration of 100 milliseconds^34^. Since the concept of signature whistles has been developed and validated primarily for bottlenose dolphins, we refer to the potentially individually distinctive calls in this study as repeated stereotyped calls. This choice reflects both the species–specific nature of the original definition and the limitations of our dataset, most notably, the absence of caller identification and detailed focal behavioral observations. Moreover, we did not constrain our analysis to signals meeting the criteria of the SIGID method, as this approach was designed specifically for bottlenose dolphin vocalizations. Instead, we adopted a broader, descriptive protocol. Repeated stereotyped whistle types were defined as a collection of whistle contours exhibiting highly similar frequency modulated patterns (e.g., Fig. 1).

### Manual classification

Whistles were visually identified as repeated stereotyped whistles by one observer (the lead author) using the spectrogram view in Raven Pro 1.6.5^51^. Spectrograms were computed using the following settings: fast Fourier transform (FFT) = 2048, Hann window, hop-size = 1024, overlap = 50%, frequency range = 0–50 kHz, time window = 3 s. Consecutive signals separated by less than 200 milliseconds and showing a continuous frequency pattern were treated as a single whistle contour. Similar whistle contours that occurred at least three times within a sequence, defined as successive whistles occurring within a 30–seconds time window within the same recording, were considered potential repeated stereotyped whistles. These sequences are hereafter referred to as “bouts”. We then selected each signal from a sequence to extract acoustic parameters, and calculated the inter-whistle intervals (IWIs) within each sequence. Once a possible stereotyped repeated whistle type was detected, the recording was scanned for further whistles of the same type, where each whistle was assigned a unique identification code and added to the database. The signal–to–noise ratio (SNR) was visually assessed for each whistle and categorised as *high* when the contour was strong and clearly distinguishable from background noise, *medium* when the whistle was clear but background noise partially overlapped or reduced contrast, and *low* if the whistle was faint and slightly visible and/or overlapped with other signals. Whistles with low SNR were included in the subsequent analyses only if the entire contour was recognisable and comparable to previously encountered medium– or high–SNR contours.

A subset of 120 stereotyped whistles that occurred repeatedly in the recordings and exhibited high or medium SNR was selected for the first step of visual categorization by the lead author. This subset of data was then categorized visually by six independent judges with prior experience in analyzing and sorting dolphin whistles. Standardized spectrograms (frequency range: 0–20 kHz; duration: 0–1.5 s) were generated for each whistle and compiled into a document. Judges sorted the whistles independently and were given the whistles in different sequential orders to minimize potential biases. They were instructed to categorize whistles based solely on the contour shape and were not provided with any information regarding the number of expected categories or whistle types. The resulting categories were clustered using a custom script in R software^52^. Inter–observer agreement was assessed using Fleiss’ and Cohen’s Kappa, to quantify both general and pairwise agreement among judges. This analysis confirmed the categorization conducted by the lead author (see results). Following this phase, the lead author categorized the remaining whistles to enable comparison between manual and automated classification methods across the whole dataset. All identified and categorized whistles were extracted from the original recordings and saved as short ‘.wav’ files.

### Automatic classification

The extracted audio files for each visually identified repeated whistle were imported into the open-source program PAMGuard^53^. We then employed the Interactive Contour Extraction tool in the Real-time Odontocete Call Classification Algorithm module (ROCCA)^54^ to extract whistle contours. ROCCA uses a peak extraction algorithm, which can be manually edited to increase extraction accuracy. Whistle contours were then categorized using ARTwarp 1.0, a MATLAB–based script^55^(version R2022b) developed for organizing tonal signals into categories based on frequency content and frequency modulation patterns^55,56^. ARTwarp uses an automated, unsupervised neural network to categorize contours based on a set threshold of similarity between contours defined as the vigilance parameter. To account for the natural temporal variation in whistle contour duration, ARTwarp also applies dynamic time warping, allowing contours to be stretched or compressed over time to better match whistles to a reference category. The following ARTwarp settings were used for the automated analysis: Warp factor level (W) = 3; Vigilance (VP) = 96.00; Bias = 0.000001; Learning rate = 0.100; Max no. of iterations = 100.

Whistle types from the visual categorisation were first analysed in a sequenced approach^65^, where each visually identified whistle type was run through ARTwarp separately using the above–specified parameters. Following Fearey et al.^23^, we assessed the agreement between the sequenced automated and visual categorisation by identifying a dominant category for each automatically categorised grouping and calculating the percentage of visually classified whistles found in that ARTWARP category.

In a second step, we conducted a global ARTwarp analysis in which all whistle contours from all visually identified categories were analysed simultaneously, using the same settings. This global categorisation approach was conducted to assess similarity in contours within and between encounters, helping to identify shared whistle types. Categories from this automatic global classification were compared with those defined visually. Total agreement was achieved when all automatically categorised contours were grouped identically to the visually categorized contours, while partial disagreement resulted from contours from each visual category being split and/or grouped in different automatically generated categories.

### Acoustic parameters and statistical analysis

ROCCA extracts a total of 50 acoustic parameters from each whistle contour^54^. From these, a subset of 7 parameters was retained to characterize the acoustic features of repeated whistles. Specifically, we considered the following: duration (W_dur_: end time – begin time), minimum and maximum frequency (F_min_ and F_max_: frequency at the lowest and highest point of the whistle contour), start and end frequency (F_start_ and F_end_: frequency at the start and end of the whistle contour), frequency range (F_range_: F_max_ – F_min_), and mean frequency (F_mean_ = (sum of all frequency values at each time point along the contour) ÷ (total number of time-frequency points)). To visualize the similarities between whistles belonging to the same category, we performed a Principal Component Analysis (PCA) using a custom script in MATLAB software.

### Ethical note

Data were collected during fieldwork carried out with permit 08/IFCN/2023 – FAU MAD issued by the Institute of Forests and Nature Conservation of Madeira (IFCN). Photo–ID is a non–invasive technique that does not require closely approaching the animals, as it uses zoom lenses. Passive acoustic monitoring is a non–invasive technique that does not involve the harassment of animals, allowing for the observation and recording of their natural acoustic behavior. Both photographic and acoustic data collection were carried out minimizing potential disturbances caused by research activities. To minimize potential disturbance when the approach and presence of the research vessel was necessary (mostly during the acoustic data collection), the engine was turned off immediately before deploying the hydrophone. Boat repositioning was conducted only when essential to ensure the quality of the data collected because animals had moved further away from the vessel.

## Results

### Photo-ID analysis

A total of 49 photographic identifications from 30 individuals were obtained from the three sampling events. Of these individuals, 17 were photographed only once and 13 on multiple times (seven individuals were photographed in two sampling events and six individuals were photographed in all three sampling events) (Table 1).

**Table 1:** Results of photo-ID analysis. Group size: total number of individuals counted visually during the sampling event. No. of calves: number of calves counted visually during the sampling event.

	20/07/2023	26/07/2023	28/07/2023
Group size	40	45	40
No. of calves	1	2	2
No. of photographic identifications	12	19	18
% of photographic identifications from group size	30	42	45
No. of individuals photographically identified in one sampling event	3	9	5
No. of individuals photographically identified in two sampling events	3	4	7
No. of individuals photographically identified in three sampling events	6	6	6

### Acoustic recordings

Details on the analysed acoustic recordings are reported in Table 2. Acoustic sampling days were analyzed separately during the initial spectrogram scanning by one observer to identify whistle contours for each day. After this, visual categorisation of all identified calls was carried out across the full dataset, independent of recording day, and assessed through an inter-observer reliability test. Only after categories were established, whistle types were compared across days to determine whether similar call types occurred on multiple sampling days. This approach allowed us to track recurring call types while accounting for day-to-day variation.

**Table 2:** Details of recordings used in the repeated stereotyped whistle analysis in the three sampling events.

Date	Initial location (Latitude; Longitude)	Tot. encounter duration (min)	No. of separate recordings during encounter	Tot. recording time (min)
20/07/2023	32.33448; – 017.05021	125	8	110
26/07/2023	32.67354; – 017.20696	243	7	56
28/07/2023	32.57956; – 017.11244	138	8	96
Total	–	506	23	262

### Manual categorisation

**Fig. 2:**
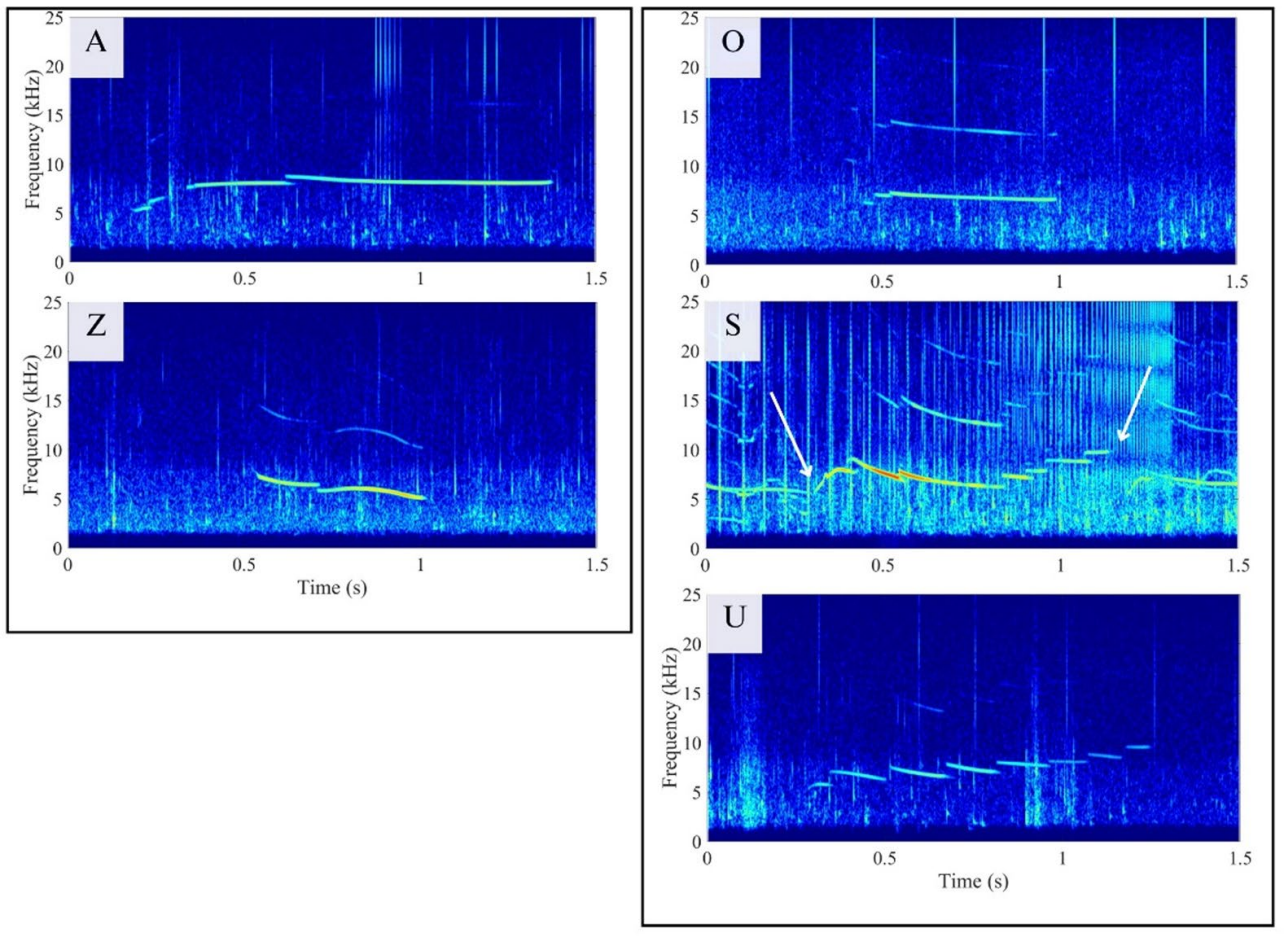
Spectrograms of exemplar contours from repeated whistle types produced by rough-toothed dolphins (*Steno bredanensis*) occurring across multiple sampling days. The left panel shows categories recorded on all three sampling days, while the right panel shows categories recorded on two of the sampling days. White arrows point at the beginning and end points of the contour of interest, as other whistles are present in the snippet. Spectrogram computed in MATLAB for illustration purposes with the following parameters: FFT = 4096, Hamming window, overlap = 90%, frequency range = 0–25 kHz, time window = 1.5 s. A bandpass filter (2–25 kHz) was applied prior to spectrogram computation, and amplitude values were omitted below − 120 dB to enhance contrast.

A total of 4928 whistles were visually identified across 262 min of recordings. Among these, 206 exhibited high signal–to–noise ratio (SNR), 1004 had medium SNR, and the remaining 3718 were classified as low SNR. The initial spectrogram scan by the lead author identified 1015 whistles that appeared to be repeated, including 116 high SNR, 477 medium SNR, and 422 low SNR whistles. In an inter-observer reliability test with six observers using a subset of 120 whistles, the inter-rater agreement was generally high, with the exception of one judge who consistently grouped multiple whistle types into broad categories, resulting in outlier behavior compared to the others. This judge was excluded from further analysis, and inter–observer reliability among the remaining five judges was assessed using Fleiss’ Kappa, yielding a value of 0.71. The five judges independently sorted the whistles into between 21 and 34 categories (mean ± SD = 26.4 ± 4.7), with pairwise agreement ranging from 60% to 82%. Our results below are based on the lead author’s classification, which showed a mean agreement of 73.3% with the other judges. The repeated whistles were assigned to 25 distinct categories, with the number of whistles per category ranging from 3 to 407 (mean ± SD = 8.8 ± 82.9). Five of the 25 visually identified categories were recorded on multiple days. Categories A and Z (Fig. 2) occurred across all three recording days. Category A contained 230 whistles, with 6 recorded on the first, 93 on the second, and 131 on the third day. Category Z contained 407 whistles, with 28 recorded on the first, 5 on the second, and 374 on the third day. Categories O, S, and U were each detected on two days: O (35 whistles on the first and 7 on the third), S (3 on the second and 5 on the third), and U (3 on the first and 64 on the third).

**Fig. 3:**
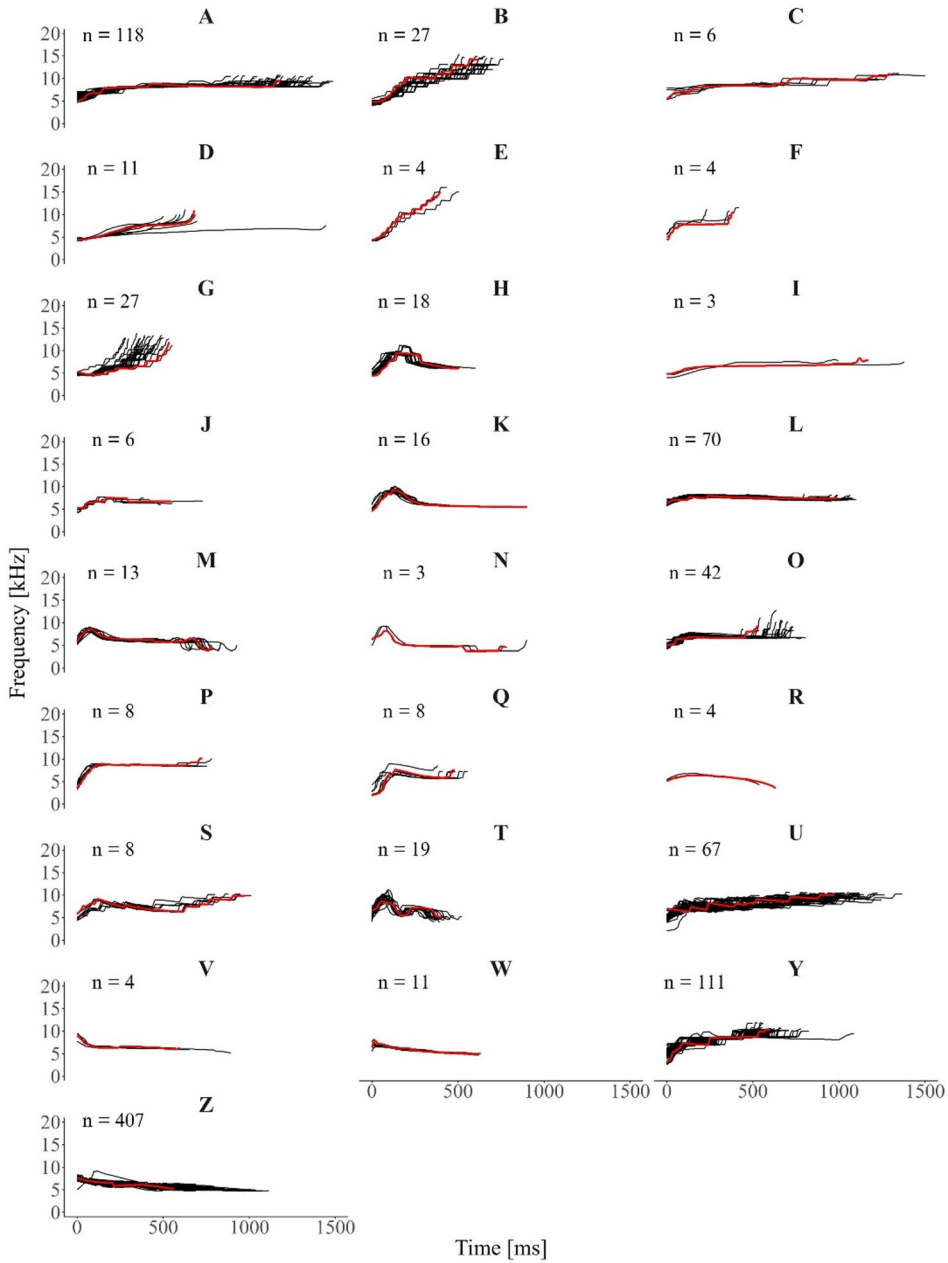
Visually identified repeated whistle contours from rough-toothed dolphins (*Steno bredanensis*). All extracted contours are in black, and a medium– or high– SNR exemplar contour for each category is shown in red. Sample size (n) is specified for each category. Bold letters above each plot indicate the assigned visual category name.

Manual classification relied on frequency contour modulation to identify repeated whistle types. While the frequency modulation of these whistles is stereotyped, signal duration can vary within each visually identified category. To illustrate this, the contours of each visually identified category are overlaid in Fig. 3. For ease of interpretation, a representative contour with a medium or high SNR was manually selected for each category and highlighted in red, overlaid on the remaining contours. Spectrograms of a high- or medium-quality exemplar for each category are provided in the Supplementary Figure S1.

**Fig. 4:**
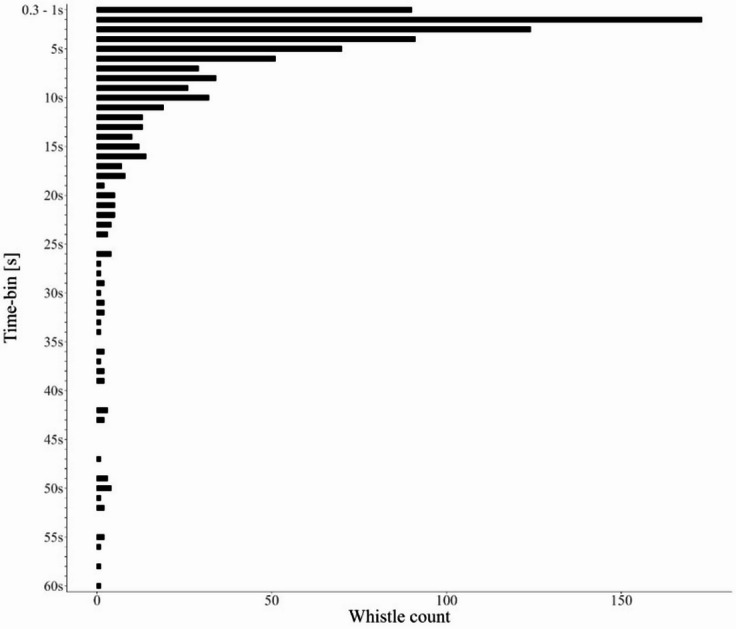
Inter-whistle interval (IWI) distribution of repeated whistle types occurring within 1 min of each other. Whistle count on the x–axis, 1 s time–bin on the y–axis, except the first time–bin encompassing 0.7s (no IWI found lower than 0.3 s).

Whistles in the same category were often produced in bouts (see bout definition in the Methods section) within the same recording sequence, ranging from 3 to 101 whistles in each bout. Inter-whistle intervals (IWIs) ranged from 0.3 s to 799 s, with only 6% of all IWIs exceeding 1 min. When considering repeated whistles with an IWI of a maximum of one minute (*n* = 883), 54.3% were repeated at intervals between 0.3 and 4 s, 27.4% between 4 and 10 s, and 18.3% were longer than 10 s (Fig. 4). The average IWI was 6.9 ± 9.2 s (mean ± SD).

### Automated categorization

We analysed the 1015 repeated whistles in several ARTWARP runs. During the sequenced automatic categorization in which visually identified whistle types were analysed individually in ARTwarp, a total of 12 out of the 25 visually identified categories reached 100% agreement with the categories produced by ARTwarp. Agreement was above 50% for the remaining categories, except for visually classified categories C, D, S and Z, for which whistle contours were almost evenly split into two main categories by ARTwarp (Table 3).

**Table 3:** Comparison of repeated whistle contours between visual categorization and the sequenced automatic method. Category: visual category assigned. Sample size: number of contours included in each visual category. Automatic categories: number of contours assigned to the same category by ARTwarp; multiple numbers separated by *“/”* indicate cases where the automatic method split the contours into multiple categories. Agreement: percentage of contours within the visual category assigned to the dominant ARTwarp grouping. The following ARTwarp parameters were used: Vigilance Parameter = 96.0 and Warping Factor = 3.

Category	Sample size (*n*)	Automatic categories (*n*)	Agreement (%)
A	118	118	100
B	27	27	100
C	6	3/3	50
D	11	10/1	90
E	4	4	100
F	4	2/2	50
G	27	26/1	96
H	18	18	100
I	3	3	100
J	6	2/4	67
K	16	16	100
L	70	70	100
M	13	13	100
N	3	3	100
O	42	17/25	59
P	8	8	100
Q	8	7/1	88
R	4	3/1	75
S	8	4/4	50
T	19	18/1	95
U	67	37/29/1	55
V	4	4	100
W	11	9/2	82
Y	111	111	100
Z	407	186/50/171	46

**Fig. 5:**
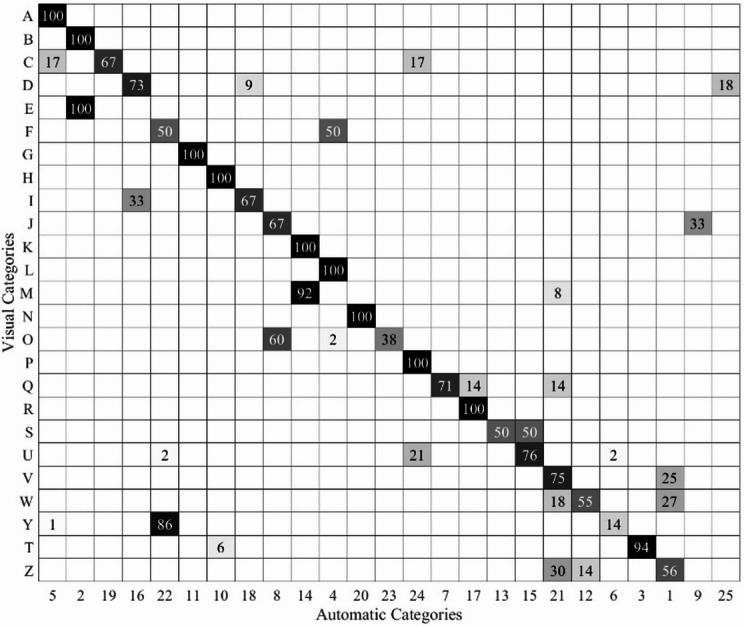
Heatmap illustrating the percentage of the global ARTwarp classification matches to visual categories. Rows represent visual categories, and columns correspond to automatic categories. Each cell shows the percentage of contours from each automatic category assigned to the visual categories. White cells represent 0% of the assigned contours to that category. The highest correspondence values are located along the diagonal, denoting the strongest matches between visual and automatic classifications. The following ARTwarp parameters were used: Vigilance Parameter = 96.0 and Warping Factor = 3.

The global automated classification, in which all whistle contours were analysed together in a single ARTwarp run, resulted in 29 categories. Most automatically identified categories included grouping or splitting of visually identified categories. Four of these categories contained only one contour and, therefore, were removed from the analysis. Of the remaining 25 categories, 9 contained all whistles of a single visually identified whistle type, of which two (11 and 20) corresponded exactly to a single visual class, containing all and only the contours assigned to that class. The remaining 16 categories consisted of a mixture in different ratios between two and five different whistle types (Fig. 5).

**Fig. 6:**
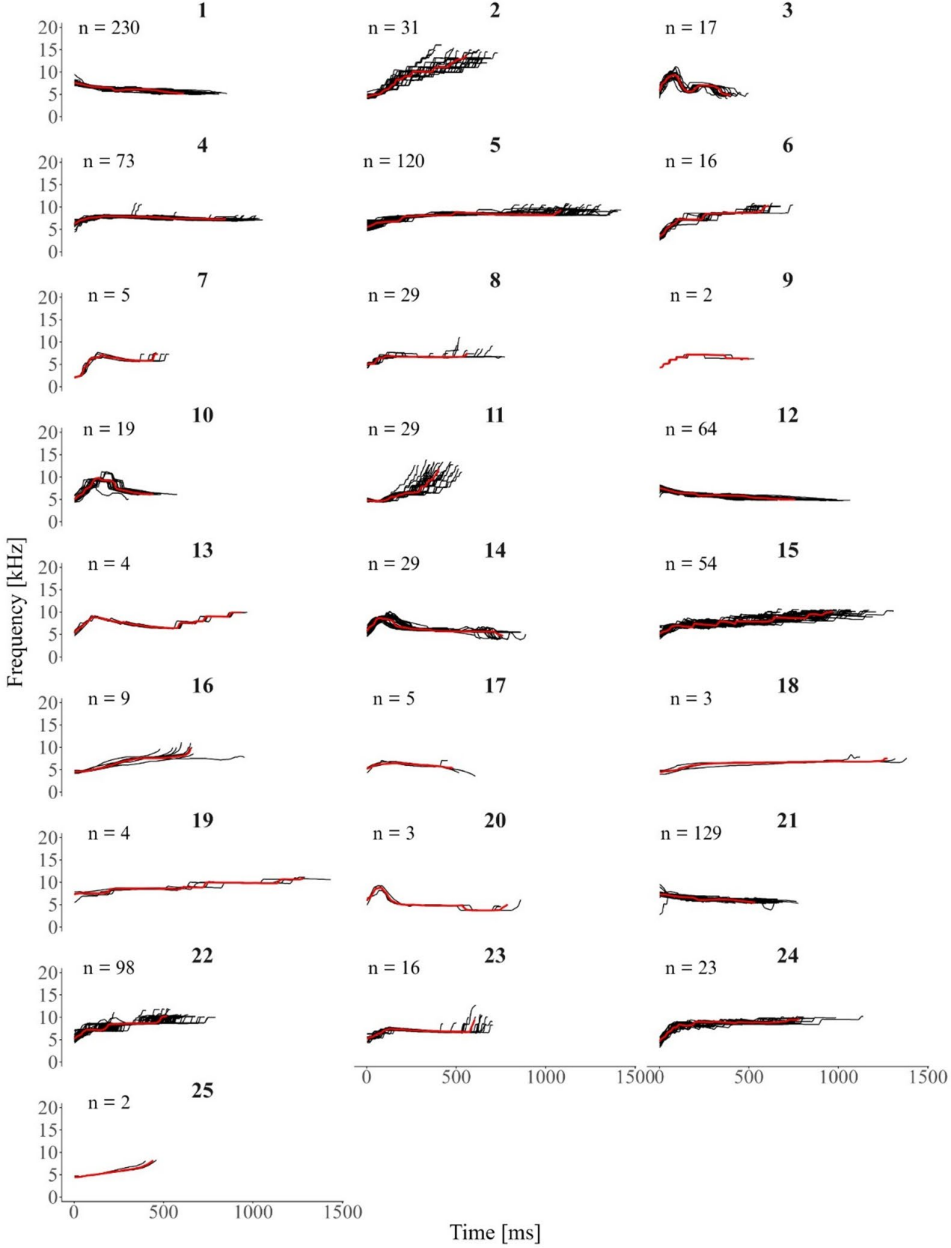
ARTwarp global automatic classification for repeated whistle types produced by rough-toothed dolphins (*Steno bredanensis*). All automatically classified contours are in black. For ease of interpretation, the reference contour generated by ARTwarp at each iteration, representing the averaged frequency modulation pattern of all contours assigned to that category, has been highlighted in red and overlaid on the remaining contours. Sample size is specified for each category. Bold numbers above each plot indicate the assigned automatic category. The following ARTwarp parameters were used: Vigilance Parameter = 96.0 and Warping Factor = 3.

Whistle contours assigned to each automatic category during the global categorisation are illustrated in Fig. 6.

### Acoustic characteristics for visually determined categories

The W_dur_ of repeated calls ranged between 0.20 and 1.28 s (mean ± SD = 0.62 ± 0.22), F_min_ from 1.90 to 7.52 kHz (5.17 ± 0.70), F_max_ from 6.33 to 16.03 kHz (8.81 ± 1.52), F_start_ from 1.90 to 9.42 kHz (6.07 ± 1.31), F_end_ from 3.38 to 16.03 kHz (7.57 ± 2.43), F_range_ from 0.56 to 11.88 kHz (3.64 ± 1.80) and F_mean_ from 4.98 to 10.33 kHz (7.09 ± 9.33). Explanations of abbreviations and detailed acoustic measurements for each whistle category are presented in the Supplementary Table S1.

All seven measured acoustic parameters were included in a PCA analysis, and an eigenvalue analysis was carried out using the Kaiser criterion, retaining only components with eigenvalues greater than 1. This criterion guided the decision to keep two PCs for further analysis, balancing dimensionality reduction with the amount of variability explained. The PCA resulted in 81.9% of variance explained by two principal components (PCs), with PC1 largely represented by F_end_, F_high_, F_range,_ F_start_ and F_mean_ (58.0%) and PC2 by F_min_ and W_dur_ (23.9%; Table 4).

**Table 4:** Contribution of each acoustic parameter of repeated whistle types in the Principal Component Analysis. Values that most contributed to each PC are in bold. Acoustic variables considered: W_dur_: duration (s), F_min_: minimum frequency (kHz), F_max_: maximum frequency (kHz), F_start_: start frequency (kHz), F_end_: end frequency (kHz), F_range_: frequency range (kHz), F_mean_: mean frequency (kHz).

	PC1	PC2
W_dur_	0.09	**0.57**
F_min_	– 0.18	**0.65**
F_max_	**0.46**	– 0.01
F_start_	– **0.41**	0.06
F_end_	**0.46**	0.10
F_mean_	**0.41**	0.35
F_range_	**0.46**	– 0.23

**Fig. 7:**
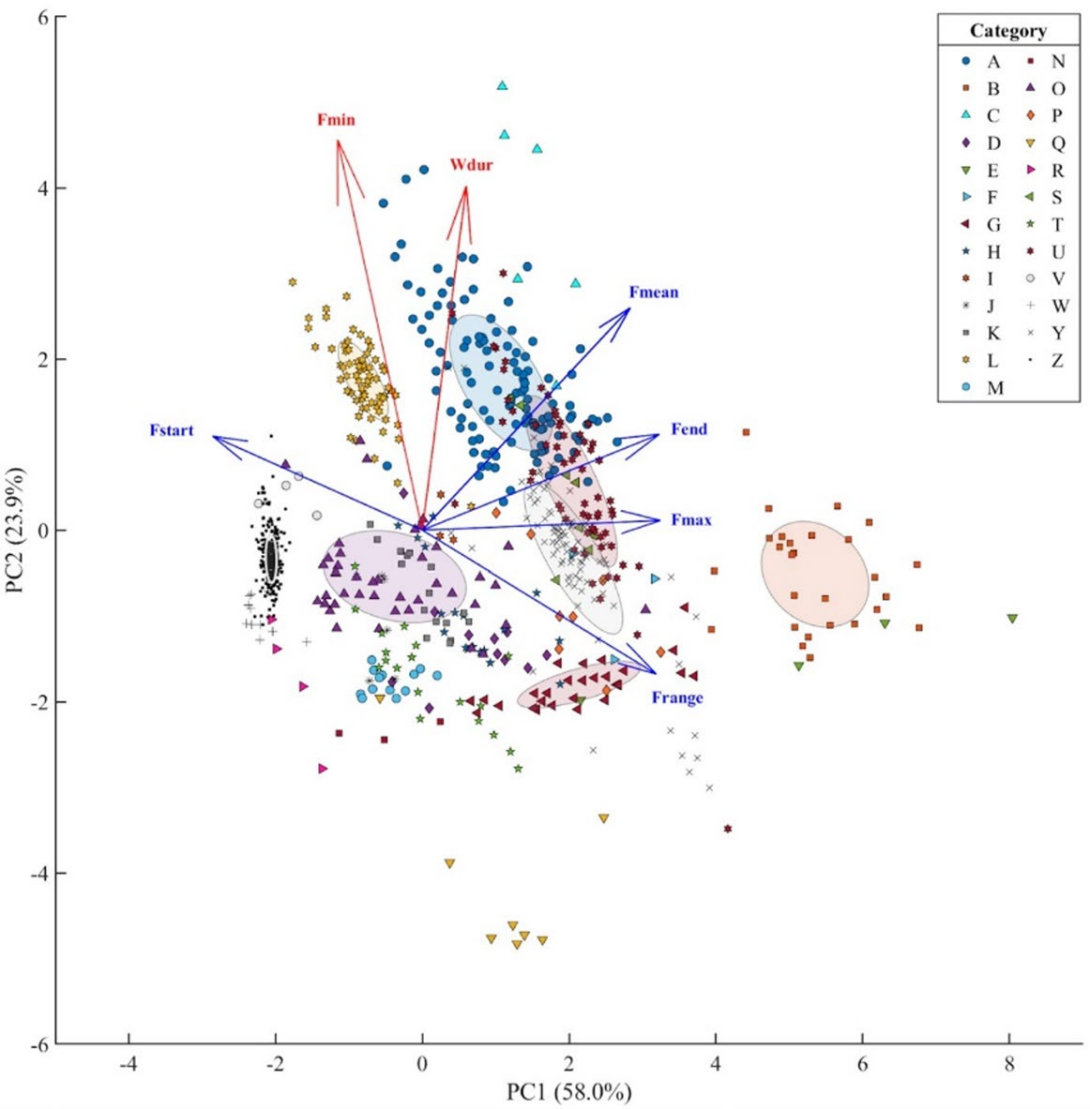
Visualization of repeated whistle types produced by rough-toothed dolphins (*Steno bredanensis*) in a two-dimensional Principal Component Analysis using acoustic characteristics. Each symbol represents a single whistle type, with symbol shapes corresponding to the visual categories as indicated in the legend. Arrows represent loadings of each parameter (in blue, the main contributors to PC1, in red to PC2). Ellipses highlight clusters of visually identified categories with more than 20 samples. Acoustic variables considered: W_dur_: duration (s), F_min_: minimum frequency (kHz), F_max_: maximum frequency (kHz), F_start_: start frequency (kHz), F_end_: end frequency (kHz), F_range_: frequency range (kHz), F_mean_: mean frequency (kHz). Percentages on axis labels indicate the percentage of variability explained by each factor.

Whistles from the same visually identified category were mostly grouped together in the PCA classification, confirming a relevant similarity in whistle characteristics within each category. However, the sparse distribution of some categories and the wide size of some ellipses suggest that the variation of acoustic parameters is likely dependent on whistle type and/or sample size (Fig. 7).

## Discussion

This study provides the first evidence of repeated whistle type production by free-ranging rough-toothed dolphins. The use of photo–ID allowed us to confirm that out of the 30 identified animals, 13 (corresponding to 43%) were photographed across the multiple sampling events. This rate of photographic recaptures, along with the close spatial and temporal proximity of the sampling events, suggests many individuals were present during multiple sampling events. However, due to the fluid (fission–fusion) social dynamics of the species^46^, it cannot be assumed that the entire group was the same. The percentages of photographic individual identifications from the group sizes during the three sampling events (of 30%, 42%, and 45%; Table 1) are consistent with findings from a population of this species in Hawai’i where at least 33% of the individuals are typically well-marked and identifiable^57^– suggesting that most of the distinctive animals in our sampling events were captured by photo–ID. Therefore, while our data cannot be interpreted as representing the vocal behaviour of an entire single group, they provide insight into the vocal behaviour of groups that include at least some recurring individuals. Although we cannot determine whether repeated calls recorded on multiple days were produced by the same individuals, this approach opens new possibilities for studying groups with varying composition and identifying the possible presence of shared whistle types across encounters. It is worth noting that while only 30–45% of the group was photographically captured each day, many of the same individuals were recaptured across events, suggesting that this acoustic dataset represents a small subset of animals repeatedly sampled. Considering that the dataset spans just three days, group-level analyses are therefore limited and should be considered hypothetical.

Across the three sampling events, 25 distinct repeated whistle categories were visually identified and subsequently assessed using an unsupervised neural network, offering novel insights into the whistle repertoire of this species. The repeated production of a relatively limited number of whistle types by rough-toothed dolphins raises questions about the function of these calls. In well-studied species such as bottlenose dolphins, repeated stereotyped whistles have been characterized in detail^7,13,18^, providing a framework for studying whistle repetition in other delphinids. Over the past 60 years, research on signature whistles in bottlenose dolphins has increased, initially focusing on isolated individuals and subsequently, with the development of the SIGID method, on wild populations^19^. While the SIGID method has so far been validated only for bottlenose dolphins, it has been tentatively applied to other delphinid species (e.g.^23,27,31–33^). The authors of these studies reported patterns consistent with those of bottlenose dolphins’ signature whistles, suggesting that individual-based vocal communication may occur broadly among delphinids, particularly in species exhibiting fission–fusion social structures. In such fluid societies, individual-specific calls may serve to advertise presence or facilitate contact with specific group members^58^. However, repeated, stereotyped calls are not exclusive to signature whistle systems. As mentioned above, in species such as killer whales, individuals also produce shared, stereotyped call types that occur frequently within groups but their defining frequency modulation pattern does not appear to be individually distinctive. These calls are often stable over time and may serve other functions such as group cohesion or identifying groups, rather than individual recognition^11,48^. This shows that repetition alone does not confirm the presence of a signature whistle.

The production of stereotyped repeated calls, potentially akin to signature whistles of bottlenose dolphins, has been suggested for rough-toothed dolphins in a recent study by Ramos et al.^33^. The authors identified a specific whistle type repeatedly produced by an isolated captive dolphin, which met the criteria from the SIGID method, potentially supporting the existence of signature whistles in this species. While such findings expand our understanding of whistle repertoires in lesser–known species, it is crucial to interpret them carefully. As already mentioned above, applying methods developed for well–studied coastal species to the calls of another species can lead to misinterpretation, especially when the ecological context and behavioral flexibility of the species are not well understood. Additionally, studies conducted on isolated or rehabilitated individuals must carefully consider how such conditions might influence behaviour, and that they may not accurately reflect natural patterns. And finally, it is unknown whether this whistle is unique to the individual or whether it is a whistle type that is also produced by other individuals. For all of these reasons, conclusions should avoid undue generalization.

In this study, repeated whistle types were investigated using multiple classification methods to improve data verification and categorization. Across all approaches, groupings of whistle types consistently emerged. Manual classification served as the baseline comparison to sequenced and global automated analysis. The results of this process provide a framework for future automated categorizations of modulated signals in this species, particularly in cases where the quantity and variability of signals make automatic classification a more efficient and practical approach. Moreover, while our study aimed to detect and classify stereotyped repeated whistles, the results suggest that repeated signals in rough–toothed dolphins exhibit inherent variability at higher levels than typically observed in species such as bottlenose dolphins, where individual identification calls are highly stereotyped^59^. Given this lower degree of stereotypy, we refer to these whistles simply as repeated whistles, as we currently lack a detailed characterization of within–individual whistle variability under natural conditions.

Manual classification was evaluated by six independent judges, following standard procedures commonly used in similar studies^50,51^. The average agreement among these judges was 71%, with pairwise agreement reaching up to 82%. While this level of agreement indicates a reasonable consistency, it is lower than what has been reported in studies of other dolphin species, where overall agreement is typically above 80%^60–62^. While manual classification is time–intensive and can be subject to observer bias, it has been shown to be highly accurate for identifying repeated stereotyped calls^60^. The lower agreement among judges compared to that of other species likely reflects the natural variability present in some repeated signals, which can complicate visual classification. This interpretation is consistent with the variability patterns observed in the acoustic parameter analysis. In particular, some categories in our dataset were more prone to disagreement. These categories were characterized by stepped contours, where small variations such as the addition or elongation of a segment resulted in changes to the overall shape. For instance, visual categories B and E were distinguished as separate by the primary observer, yet some judges grouped them together. A closer inspection revealed that the main difference lay in the elongation of a central segment, which significantly altered the perceived contour (Supplementary Fig. S2). Similarly, categories U and S created disagreement, as an elongation of one central segment might have transformed an upsweeping signal into an upsweep–downsweep–upsweep pattern. Categories A and C also showed relatively low agreement, likely because an additional segment modified the visual perception of the contour. While the lead author classified these as distinct types based on the added segment, some judges interpreted it as a natural variation and merged the two categories. These potential matches and splits were discussed only after the inter-observer reliability test was completed, ensuring that no prior bias influenced the observers during classification. Interestingly, other categories (e.g., L and O; Fig. S2) also showed within-category variability, but did not result in the same level of disagreement, likely because the variations did not meaningfully alter the overall contour perception.

Stereotyped whistles of bottlenose dolphins are known to exhibit variability, particularly in overall duration and/or the length of specific segments^7,59,61,63^. However, the highly stepped nature of rough-toothed dolphin signals may make these variations more difficult for observers to detect or consistently interpret. A slight change in a single segment can significantly affect the overall shape, leading to classification challenges. While all judges in this study had prior experience with dolphin whistle classification, most of that experience came from work on other delphinid species, such as bottlenose and common dolphins. The whistles of these species are typically characterized by smoother contours, often featuring multi-looped structures where the variability in duration is due to the addition or omission of loops, rather than changes in discrete segmental structure. Additionally, the limited frequency modulation of rough-toothed dolphin whistles as compared to other species constrains contour diversity and can inflate apparent similarity between calls. This makes direct comparisons to the highly modulated whistles of species such as bottlenose dolphins more challenging and underscores the need to interpret structural patterns within the species-specific acoustic context. Therefore, it is possible that rough–toothed dolphin whistles pose additional categorization challenges, both due to their intrinsic structural characteristics and the relatively limited research focusing on their contour variability. Future studies should quantify the natural within–type variability of these signals to better understand the range of variation in repeated whistle types. Since it is unknown which acoustic features rough–toothed dolphins use to recognize their own whistles, documenting this variability is a crucial first step in understanding their communication. This will allow comparisons with species that produce clearly stereotyped whistles, providing insights into the communicative strategies of this species.

The more concordant automated classification occurred when visually identified whistle contours were validated using a sequenced, type–by–type classification in ARTwarp (Table 3). Visual categorisation relied on spectrogram representations before contour extraction, leveraging auxiliary information such as signal intensity. Similarly, the global classification performed by ARTwarp showed relatively low agreement with visual categorisation. The analysis resulted in 25 automatic categories, some of which combined whistles from multiple visual types (Fig. 5). In the global analysis, all 1015 whistle contours were analysed simultaneously, whereas the sequenced approach limited the number of whistles analysed concurrently (to a maximum of 407 for visual category Z) as well as the amount of variation in the whistles analyzed concurrently. This reduction in overlap likely contributed to the more concordant categorisation outcomes observed between the sequenced and visual classification. Examination of the automatic categories showed that contours grouped together generally shared similar modulation patterns, such as upsweeps, downsweeps, or stepped contours. However, some visual categories were distributed across multiple automatic categories, reflecting variation within whistle types and the difficulty of capturing all subtle differences in a single category (Fig. 5). Overall, the automated global analysis captured broad patterns of whistle similarity while also highlighting areas of variability within and between visually defined types.

Interestingly, the visual categories that showed disagreement among human judges were often grouped together in the automatic classification. This automatic merging of multiple visual types by ARTwarp primarily reflects contour similarity and should not be interpreted as evidence of shared functions. However, it suggests that the algorithm may have captured underlying acoustic similarities that were not consistently recognized during manual categorization. For instance, contours from visual categories U and S (Supplementary Fig. S2), which sparked debate among judges due to the elongation of a central segment, were both predominantly grouped within automatic category 15. Similarly, categories B and E, where disagreement centered on whether a middle segment’s lengthening could justify separate classification, were entirely contained within automatic category 2. A comparable pattern was seen with categories A and C, which were largely classified under automatic category 5, again indicating that subtle structural variations may have been interpreted differently by observers but treated consistently by the algorithm. Rather than implying that whistle structure is stable, this consistency likely reflects the influence of the dynamic time warping step in the automatic method, which allows temporal variation between whistles of the same type. Bottlenose dolphins regularly expand and shorten whistle sections within the same whistle type and this might also be the case for other species. ARTwarp’s ability to allow for such variation likely led to a higher consistency in classification that was more difficult to achieve by visual classification alone. In this sense, the discrepancies between visual and automatic classifications may highlight the added value of automated approaches in revealing underlying structures that could be missed in manual analyses and result in more consistent categorization that can be compared more effectively across studies.

This is the first description of the temporal occurrence patterns of repeated whistles in wild rough-toothed dolphins. By analyzing whistles repeated at least three times within a single bout, IWI was found to peak most frequently between 1 and 2 s (19.6%; Fig. 4). However, a notable proportion of calls occurred at shorter (0.3–1 s: 10.2%) or longer intervals (2–10 s: 51.8%; Fig. 4). These values fall within the range of IWIs reported for signature whistle repetitions in bottlenose dolphins, where most were within a 5–10 s window but showed variability^34^. Such variability has been linked to contextual changes, including separations and (re)unions, highly arousing events, variations in group size and composition, and environmental conditions^7,64–66^. Therefore, the observed variability may similarly reflect flexible emission rates associated with different social or environmental conditions. However, without concurrent behavioral observations, the functional significance of these temporal patterns remains speculative.

Nearly 5000 whistles were identified in the analyzed dataset. Of these, 1015 were classified as repeated (including high–, medium–, and low–SNR signals). The repeated whistle count includes all whistles for which the contour could be confirmed (see Methods), representing roughly 20% of the dataset. While some repeated whistles may have been missed due to low–quality signals that prevented contour verification, it appears that repetition is a recurrent but not predominant feature of rough–toothed dolphin whistle repertoires. Notably, no repeated whistles overlapped with similar signals in time, which could suggest that they were emitted by the same individual, although this cannot be confirmed without simultaneous visual or acoustic tracking. It is also possible that some repeated calls reflect whistle matching or copying between individuals, as has been observed in bottlenose dolphins^67,68^, though the present data do not allow this to be tested. Alternatively, repeated signals could reflect group-level call sharing, with the variability of signals arising from individual variability in whistle production and/or different social or environmental contexts within the group. While the lack of direct behavioural observations precludes a definitive functional analysis, the presence of repeated whistles, even at moderate levels, is consistent with the hypothesis that signal redundancy may contribute to communication within dynamic groups. In theory, repetition can enhance detectability by broadcasting identity and information and may reduce the risk of masking in complex acoustic environments dominated by conspecific biophony^5,69^. Given that rough–toothed dolphins form groups composed of several tight subunits of individuals swimming in close synchrony, but with these subunits often separated by tens of meters from other subunits or solitary group members^47^, such repeated signals could potentially facilitate individual identification and coordination across dispersed group members. As hypothesized for other species, it is possible that structured associations such as mother–calf pairs may benefit from localized acoustic “sheltering,” facilitating information transmission and cohesion within broader group movements^70^. Short, repetitive signals produced at regular intervals could further aid individual identification and localization, even if repetition is not the dominant communicative strategy. Additionally, the limited number of distinct repeated whistle types detected may reflect the observed dispersed spatial arrangement of large groups of animals around the boat and their movement relative to the hydrophone, which could have affected the detectability of less frequent or lower–amplitude call types. It may also be noted that no multi–looped repeated whistle types were observed in this study. This aligns with findings from other wild populations of rough-toothed dolphins but contrasts with the captive study^33^, where 23.6% of potential signature whistles were double or triple–looped. This discrepancy may suggest that multi-looped signals could be linked to specific contexts such as stress or isolation observed in captive settings. Alternatively, such signals may represent acoustic adaptations for transmitting in noisy environments, as proposed by Probert et al.^71^, which could explain their occurrence in coastal water species^32,62^. In contrast, pelagic delphinid populations, such as the rough–toothed dolphins in the present study, may rely on short, repeated signals to avoid the masking of important acoustic features by conspecifics vocalising in the same frequency band while in large groups, while not necessarily being affected by the acoustic clutter and reflectivity of shallower habitats. However, as our dataset lacks concurrent behavioral observations and a larger longitudinal sample size, these interpretations of biological function remain tentative and should be interpreted with caution.

The acoustic characteristics of the whistles described here are consistent with those reported in other populations of rough-toothed dolphins^40,43–45^, indicating that repeated signals generally fall within the species’ typical range. However, further analysis of this population’s vocal repertoire is needed to confirm possible acoustic differences between repeated and non–repeated signals.

As noted above, stereotyped whistles produced by bottlenose dolphins can exhibit variations associated with behavioral context or arousal states^20,63^. In our dataset, PCA analysis showed that whistles within visually identified categories tended to be acoustically more similar to each other than to those in other categories (Fig. 7), supporting the presence of structured and recurrent call types. Clustering patterns, however, varied, suggesting that some of the observed variability might reflect sample size rather than acoustic instability and, at the same time, that the natural variation observed in repeated signals likely contributes to spreading within categories, reflecting subtle changes in the acoustic structure of similar calls rather than differences in signal type.

A key limitation of trying to identify individually distinctive calls in wild marine mammals, especially those living in cohesive groups, is the inability to identify the caller. However, none of the repeated calls analyzed overlapped temporally with similar contours, suggesting that certain call types may be produced by particular individuals, except in cases of whistle copying^67^, which has not yet been assessed in this species. This dataset provides a first glimpse into repeated whistle types in wild rough-toothed dolphins; however, it is limited in both temporal scope and the variability of individuals sampled. A larger and more diverse dataset is required to advance meaningful interpretations regarding the group-level occurrence and function(s) of repeated call types for this species. On the other hand, captive studies can confirm and investigate repeated calls on the individual-level, although their artificial setting may limit the generalization of findings to the natural environment.

To conclude, this study provides evidence for the production of repeated whistle types in free–ranging rough–toothed dolphins. These calls were identified through a combination of manual and automated classification methods. The automated approach proved effective in assessing whistle similarities while accounting for temporal variations. In contrast, visual classification remains more sensitive to fine contour modulation patterns and subtle structural variations as compared to automated categorization using ARTwarp. These findings highlight the complementary strengths of both methods and support their combined use for a more comprehensive and reliable characterization of dolphin whistle repertoires. Future works should investigate supervised computer classification approaches to explore whether combining methods leads to improved categorization. The degree of stereotypy in rough-toothed dolphin whistle types presented here was lower than that observed in some well-studied delphinid species, indicating species–specific differences in vocal behavior and/or variation within the signals themselves. These repeated signals could arise from individual-specific calls, group-level call sharing, or context-driven variability, such as responses to environmental or social cues. For a species inhabiting mainly open–ocean environments and forming large and sometimes dispersed groups, the use of repeated signals could facilitate cohesion, coordination, or information exchange among conspecifics. However, since our dataset lacks detailed behavioral or contextual data, these potential functions remain speculative. Future research should combine acoustic recordings with detailed behavioral observations, high-resolution acoustic archival tags and/or acoustic localization using multiple hydrophones to determine which animals produce repeated whistles, under what circumstances, and how receivers react to these signals. Collecting larger and more diverse datasets will be essential for testing hypotheses regarding the function, occurrence, and social relevance of repeated call types, ultimately contributing to a more comprehensive understanding of communication in this species and providing broader insights into delphinid vocal communication.

## Supplementary Information

Below is the link to the electronic supplementary material.

Supplementary Material 1

Supplementary Material 2

Supplementary Material 3

## Data Availability

The datasets generated during the current study are available in the Supplementary Information of this manuscript. The raw acoustic data analysed during this study are available from the corresponding author upon request.
