# Supplementary material for: Identification and classification of repeated whistle types from free-ranging rough-toothed dolphins (Steno bredanensis)
Source: Sci Rep. 2026 Mar 20;16:14327. doi: 10.1038/s41598-026-44853-2 (PMC13144356; doi:10.1038/s41598-026-44853-2)
Supplement: Supplementary file 3 — Supplementary Material 3 [file 41598_2026_44853_MOESM3_ESM.pdf]

SUPPLEMENTARY MATERIALS

Identification and classification of repeated whistle types from free-ranging rough-toothed dolphins (*Steno bredanensis*)

Laura Redaelli [1,2,3] *, Vincent M. Janik [4], Filipe Alves [1,2], Julie N. Oswald [4], Marc Fernandez [1,2], Eliette Hamard [1,2], Laela S. Sayigh [5], Manuel E. dos Santos [3], Ana Dinis [1,2], Francesco Caruso [6]

[1] MARE – Marine and Environmental Sciences Centre/ARNET – Aquatic Research Network, Regional Agency for the Development of Research, Technology and Innovation (ARDITI), Funchal, Madeira, Portugal

[2] Faculty of Life Sciences, University of Madeira, Funchal, Portugal

[3] MARE – Marine and Environmental Sciences Centre/ISPA – Instituto Universitário, Lisbon, Portugal

[4] Sea Mammal Research Unit, Scottish Oceans Institute, School of Biology, University of St. Andrews, Fife, UK

[5] Biology Department, Woods Hole Oceanographic Institution, Woods Hole, MA, USA

[6] Department of Marine Animal Conservation and Public Engagement, Stazione Zoologica Anton Dohrn, Naples, Italy

* Corresponding author: laura.redaelli@mare.arditi.pt

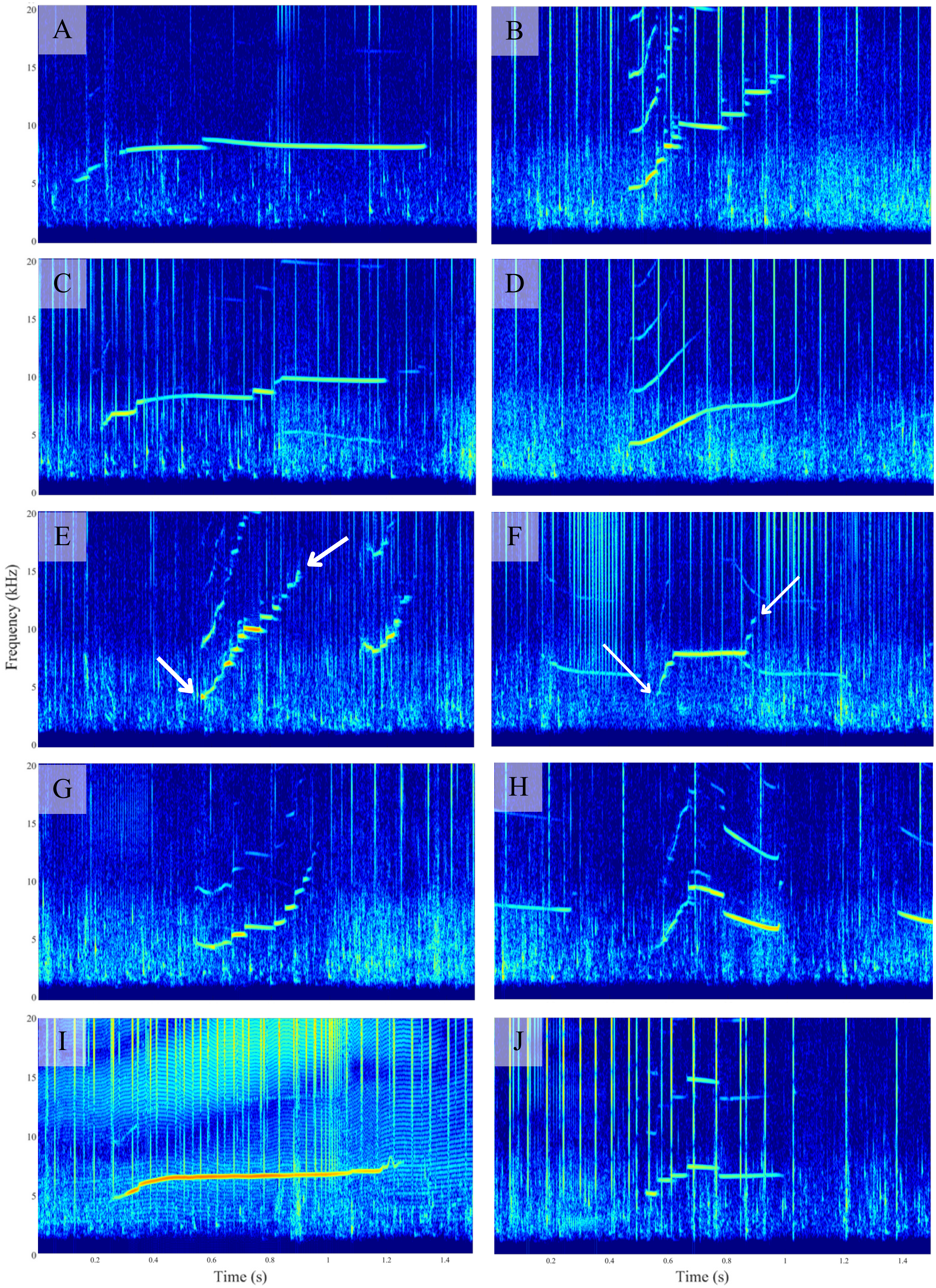
A
B
C
D
E
F
G
H
I
J
Frequency (kHz)
Time (s)
Time (s)
0
5
10
15
20
0.2
0.4
0.6
0.8
1
1.2
1.4

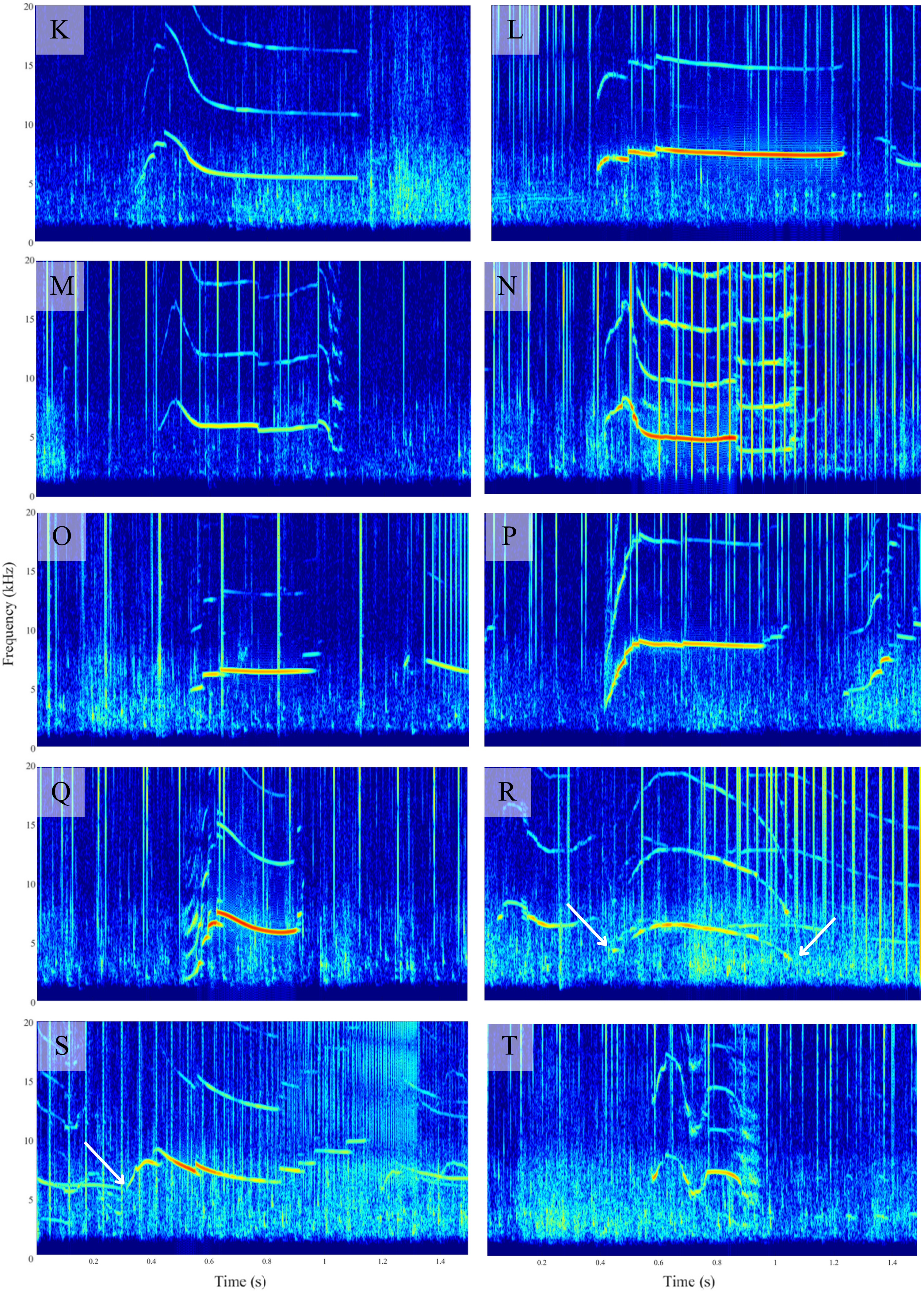
K
L
M
N
O
P
Q
R
S
T
Frequency (kHz)
Time (s)
Time (s)

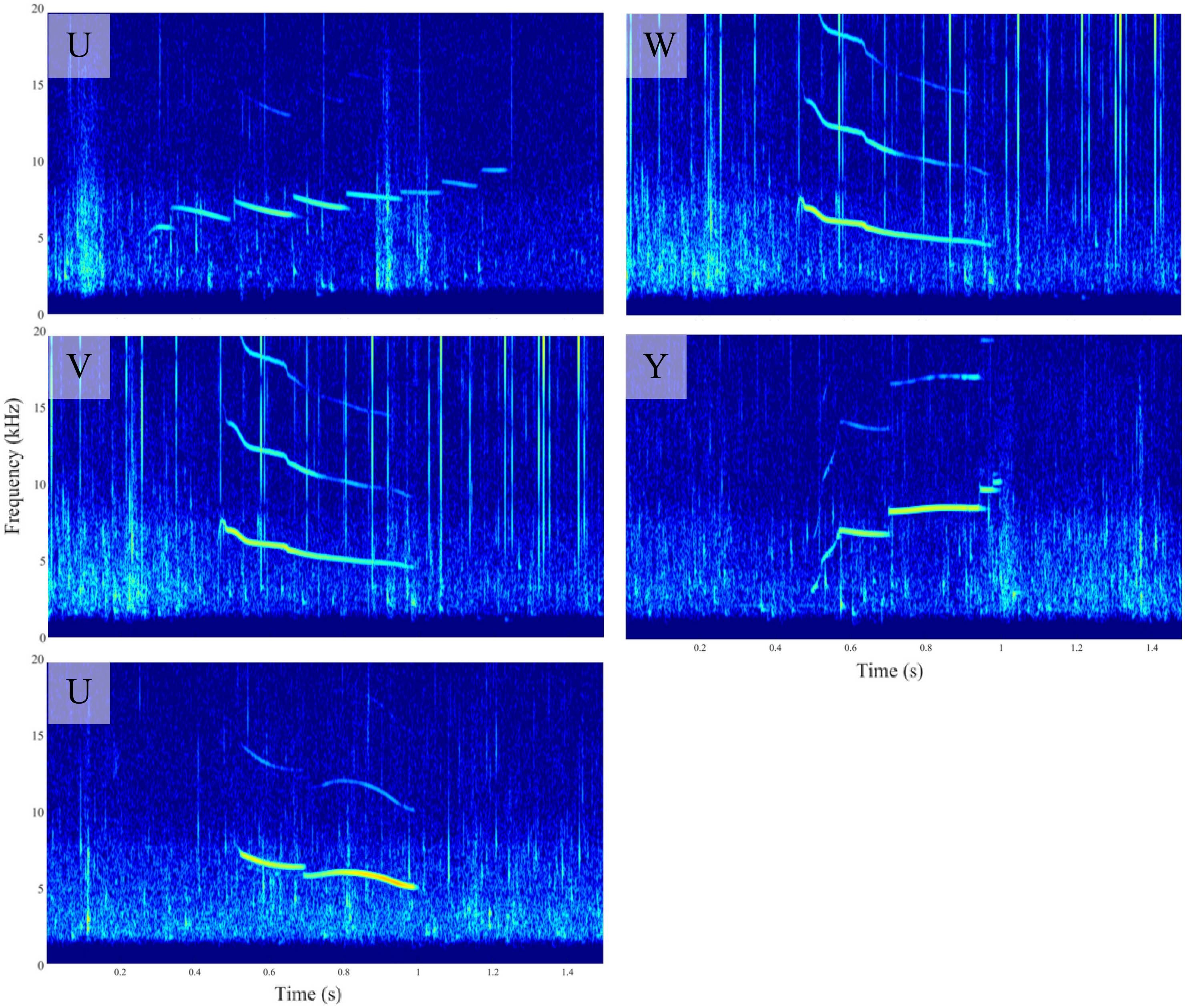

Figure S1. Spectrograms of a high- or medium- quality representative of visual categories.
Spectrograms refers to contours presented in the visual classification (figure 2 in the manuscript). Spectrograms computed in MATLAB for illustration purposes with the following parameters: FFT = 4096, Hamming window, overlap = 90%, frequency range = 0 – 20 kHz, time window = 1.5 seconds. Frequency on the y-axis (0–20 kHz), time on the x-axis (seconds). A bandpass filter (2–25 kHz) was applied prior to spectrogram computation, and amplitude values were clipped below –120 dB to enhance contrast.

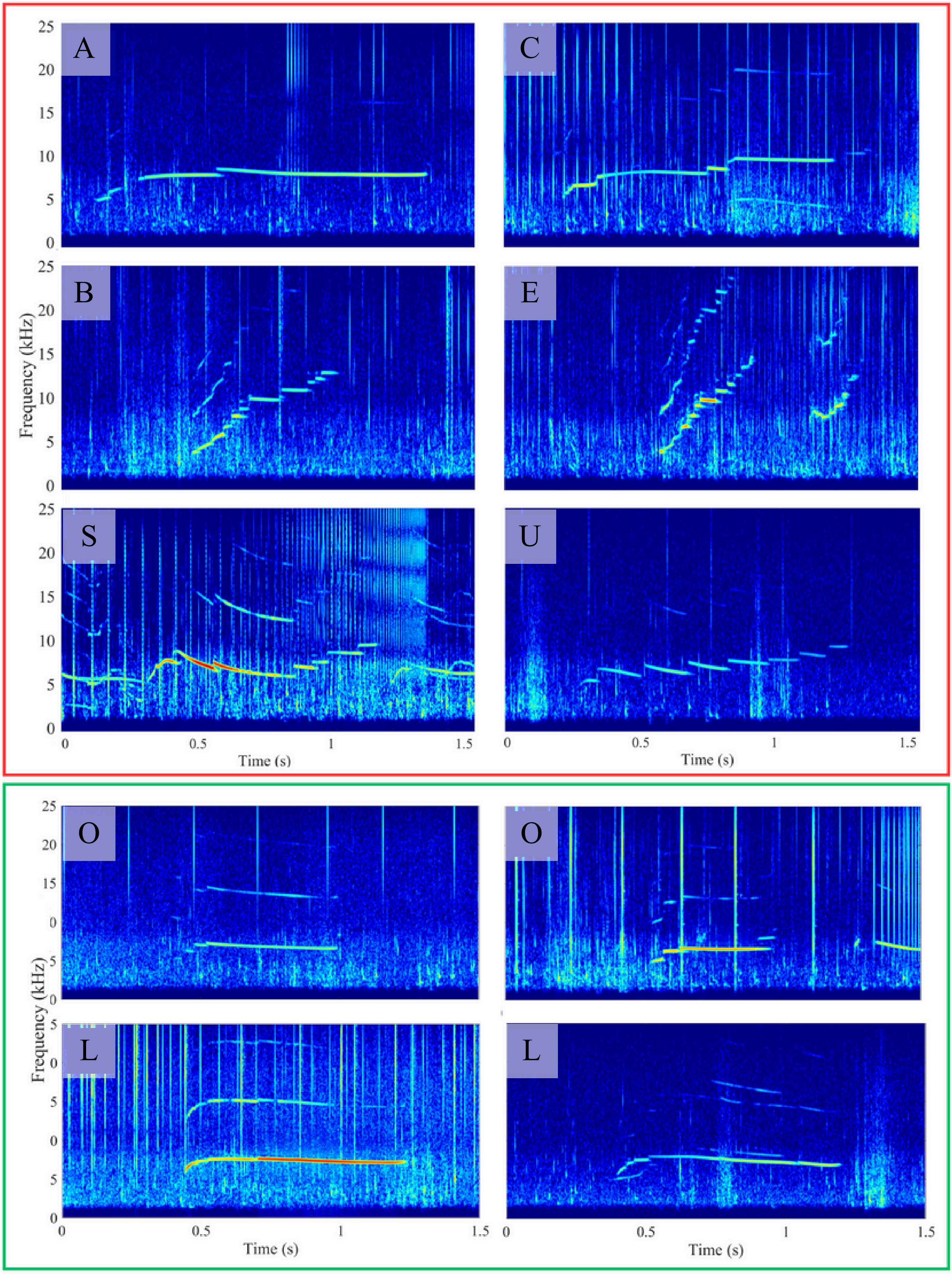

Figure S2. Spectrograms of visual categories that resulted in lower agreement among the five judges. The top panel shows pairs of contours (one per row) that were inconsistently grouped or matched by different judges. The bottom panel shows contours that exhibited some variability but did not generate substantial disagreement. Spectrograms computed in MATLAB for illustration purposes with the following parameters: FFT = 4096, Hamming window, overlap = 90%, frequency range = 0 – 25 kHz, time window = 1.5 seconds. Frequency on the y-axis (0–25 kHz), time on the x-axis (seconds). A bandpass filter (2–25 kHz) was applied prior to spectrogram computation, and amplitude values were clipped below –120 dB to enhance contrast.
